# Editorial: Exercise and biomechanical intervention in the prevention, management and rehabilitation of neuro-musculoskeletal disorders

**DOI:** 10.3389/fphys.2023.1260147

**Published:** 2023-07-31

**Authors:** Qichang Mei

**Affiliations:** ^1^ Faculty of Sports Science, Ningbo University, Ningbo, China; ^2^ Research Academy of Grand Health, Ningbo University, Ningbo, China; ^3^ Auckland Bioengineering Institute, The University of Auckland, Auckland, New Zealand

**Keywords:** sport injury, spine biomechanics, lower extremity, ACL, foot and ankle, computational biomechanics, rehabilitation

Neuromusculoskeletal biomechanics has been a popular tool for understanding the disorders of the human motor system in everyday and sports-specific activities and clinical practice. Recently, the interdisciplinary fusion of techniques has been widely employed in the prevention, management, and rehabilitation of neuromusculoskeletal disorders. With the rapid development of technologies over the past decades, cross-platform challenges have been overcome, specifically from subject-specific to population-based studies, from exercise intervention and rigid dynamics to continuum mechanical loading, training adaptability, and local tissue damage. The interest in promoting neuromusculoskeletal health with either experimental or computational techniques, or even combining the approaches, would offer promising plausibility for the prevention and rehabilitation of motor disorders or diseases.

With this in mind, the editorial team organized this Research Topic (RT) to serve as a compendium of the above-mentioned techniques for understanding neuromusculoskeletal disorders. A total of 20 studies, including 16 original articles and four review articles, were collected for publication in the current Research Topic, entitled “*Exercise and Biomechanical Intervention in the Prevention, Management, and Rehabilitation of Neuromusculoskeletal Disorders*”.

The exercise intervention studies covered gait retraining to facilitate arch function Shen et al. and found that a 12-week forefoot running program together with foot core exercises increases arch height and hallux strength. Prolonged static stretching can reduce quadriceps stiffness (particularly the rectus femoris and proximal patellar tendon) and improve knee range of motion Zhu et al. As with the Achilles tendon, a systematic review was conducted and found that eccentric strength training can improve power generation, and concentric strength training would improve balance and postural control for the rehabilitation of mid-portion Achilles tendinopathy (AT) Kim et al. Meanwhile, for a specific cohort, a systematic review of the physical exercise would improve muscle strength and reduce the pain in military pilots with neck and shoulder pain Heng et al. A combination of conventional and functional strength training showed benefits in the prevention of back pain in emergency responders Kong et al.

In order to address foot posture changes during running (Mei et al., 2019), an orthotic intervention for runners with pronated foot posture was developed to prevent potential running-related injuries (Cheng et al.; Zhang and Vanwanseele), and the center of pressure path was altered with redistributed plantar pressure loads and reduced forefoot motion in the pronated foot. Kinesio-type (KT) interventions have been used as an acute strategy to assist postural control after fatigue in athletes with functional ankle instability (FAI) Li et al. showing improved dynamic postural control and reduced risk of re-injury. ACL (anterior cruciate ligament) injury, with high risks in the knee joint, has attracted excellent studies, specifically the development of a Ligs digital arthrometer to investigate mechanical stresses and rupture mechanisms Li et al. In terms of functional recovery, an *in-vivo* evaluation of multiplanar kinematics in the knee joint during hopping activities Zhou et al. Other well-designed interventions involving strength training, proprioception, and sensation were conducted to support rehabilitation after ACL reconstruction Hu et al.; Ma et al. as different strategies for return-to-sport in patients.

Considering the pivotal role of the spine and trunk in the entire motor chain, any deformity, such as Adolescent Idiopathic Scoliosis (AIS), would lead to compensation in postural stability and gait performance Liu et al. showing the altered center of pressure trajectory patterns, pelvic motion, and gait spatiotemporal features. Computational finite element (FE) modeling, which has the advantage of being non-invasive and subject-specific, has been conducted mainly in spinal (thoracic or lumbar) disorders, with an evaluation of different fixations in the upper instrumented vertebra Pan et al. multiple pelvic screws and multirod construct Yang et al. and pedicle screw and cortical bone trajectory fixation Pei et al. The digitization of the different designs and the efficacy feedback from computational modeling could provide important clinical guidance prior to surgical correction. Furthermore, computational FE analysis for knee arthroplasty was systematically introduced and discussed, covering aspects of design and material selection and understanding of kinetic alignment and unilateral and total knee arthroplasty Zhang et al.

Neuromuscular control plays a crucial role in motor control during the execution of various tasks. Meanwhile, the disorders in the neural system, which were assessed and diagnosed using muscle synergies, have been systematically reviewed in the literature, covering the methodological Research Topic and clinical implications Beltrame et al. The handgrip task evaluation was measured with a novel sensor-embedded digital device to monitor upper extremity function and provide clinical implications for stroke and age-related function decline Ma et al. Hanger reflex intervention was performed to stimulate cervical muscle activation for rehabilitation Wang and Liu, reporting that cervical activities were substantially improved, with bilateral hanger reflex intervention showing a greater effect than unilateral hanger reflex intervention.

This Research Topic published the latest approaches to exercise and biomechanical intervention for neuromusculoskeletal disorders and several findings of practical and clinical importance. Due to the popularity of this Research Topic, we are currently organizing a second volume as a compendium for the multidisciplinary community, with a particular interest in the application of data-driven and Digital Twin (DT) technologies to understand neuromusculoskeletal disorders.
